# Impact of Frailty on Post-Treatment Dysphagia in Patients with Head and Neck Cancer

**DOI:** 10.1007/s00455-024-10754-7

**Published:** 2024-08-28

**Authors:** Javier Hurtado-Oliva, Hans Paul van der Laan, Julius de Vries, Roel J. H. M. Steenbakkers, Gyorgy B. Halmos, Inge Wegner

**Affiliations:** 1 Department of Otorhinolaryngology, Head and Neck Surgery, University Medical Center Groningen, University of Groningen, Hanzeplein 1 , PO box 30.001, Groningen, 9700RB the Netherlands; 2 Departamento de Fonoaudiología, Facultad de Medicina, Universidad de Chile, Santiago, Chile; 3 Department of Radiation Oncology, University Medical Center Groningen, University of Groningen, Groningen, the Netherlands

**Keywords:** Frailty, Head and neck cancer, Geriatric assessment, Dysphagia, Toxicity

## Abstract

In the rising and frail head and neck cancer (HNC) population, geriatric assessments are crucial. Frail HNC patients often experience dysphagia. The coexistence of dysphagia and frailty presents complex health challenges, however, there is limited evidence on the prognostic value of frailty on post-treatment dysphagia. This study explores the relationship between pre-treatment frailty status and post-treatment dysphagia in HNC patients. A retrospective data analysis from the OncoLifeS data-biobank at the University Medical Center Groningen of 242 patients diagnosed with HNC between 2014 and 2016. The study involved several physical, functional and psychological pre-treatment geriatric assessments, and frailty screening using the Groningen Frailty Indicator (GFI) and the Geriatric-8 screening tool (G8). Outcome measures were swallowing-related quality of life (HNSW-QoL) and toxicity-related dysphagia evaluations (CTCAE-D) at 3, 6, 12 and 24 months. Linear mixed-effects models assessed factors associated with HNSW-QoL and CTCAE-D. Frail patients consistently reported worse HNSW-QoL and CTCAE-D than non-frail patients over time, with symptoms increasing at 3 months, but gradually decreasing by 24 months. Frailty status (G8 or GFI) was a significant predictor for lower HNSW-QoL (β = 11.770 and 10.936, both *p* < 0.001), and lower CTCAE-D (β = 0.245, *p* = 0.058; β = 0.331, *p* = 0.019), respectively. In this study, frailty was found to be associated with a worse of swallowing-related quality of life, and with increased toxicity-related dysphagia. These findings provide insights for the identification of HNC patients at higher risk of post-treatment swallowing-related issues, and offer opportunities for optimizing their post-treatment swallowing outcomes.

**Supplementary Information:**

The online version contains supplementary material available at 10.1007/s00455-024-10754-7.

## Introduction

Population aging is a global concern, projected to continue for the next 30 years and to result in nearly 2 billion people over 65 by 2050 [1]. This phenomenon rises the incidence of older patients with head and neck cancer (HNC) as well [2, 3].

Patients with HNC have a high risk for physical, functional and psychosocial deterioration, and as a consequence are considered a vulnerable population [4], being frailer than patients with other solid malignancies [5]. The Comprehensive Geriatric Assessments (CGA) is currently the gold standard for diagnosis of frailty, describing the biological state of increased vulnerability and susceptibility to adverse events following a stress event [6]. The identification of geriatric deficits and the diagnosis of frailty in this population through the CGA, involving physical, functional, and psychological status, can improve the development of tailored treatment plans and subsequent treatment outcomes [7, 8].

Dysphagia is a highly relevant adverse outcome in patients with HNC, since up to 75% of the patients with HNC experience complaints before, during, and after oncological treatment [9, 10]. The nature and severity of dysphagia in patients with HNC may vary according to tumor site, tumor size, and the type of oncological treatment [9, 11]. Dysphagia is also currently considered as a geriatric syndrome, because of its high prevalence among older people, association with comorbidities, poor prognosis, and need for a multidisciplinary approach [12].

In this scenario, the coexistence of dysphagia and frailty status in patients with HNC results in a complex health condition, as they can be considered risk factors for each other at the same time. Currently, the relation between geriatric deficits and post-treatment dysphagia in HNC has not been studied yet. The aim of the study was to explore the relation between pre-treatment frailty status and post-treatment dysphagia in patients with HNC.

## Materials and Methods

### Ethical Considerations

Data from the present study were retrospectively collected from the Oncological Life Study (OncoLifeS), a prospectively collected oncological data-biobank approved by the Medical Ethical Committee of the University Medical Center Groningen (UMCG) [13]. OncoLifeS is registered in the Trial Register of the Netherlands under registration number NL7839. The study protocol was approved by the OncoLifeS scientific board. All patients enrolled provided their written informed consent.

### Study Population

All patients diagnosed with a primary or recurrent mucosal malignancy of the lip and oral cavity, nasopharynx, oropharynx, hypopharynx or larynx, or lymph node metastases of an unknown primary cancer were included. Included patients were diagnosed at the Department of Otorhinolaryngology and Head and Neck Surgery, and the Department of Oral and Maxillofacial Surgery of the UMCG between October 2014 and April 2016. The cohort included patients of any age that underwent curative treatment through surgery with or without postoperative (chemo)radiotherapy, or primary (chemo)radiotherapy. Treatment planning was discussed at the multidisciplinary head and neck tumor board of the UMCG. Treatment was applied according to national and international guidelines using intensity-modulated radiotherapy with or without concurrent chemotherapy. In most cases, platinum-based chemotherapy was used. Less frequently, cetuximab was used. Patients with palliative treatment intent and patients who did not provide written informed consent were excluded.

### Data Collection

Patient, tumor, and treatment characteristics were obtained from the OncoLifeS data-biobank and electronic medical records. Tumor staging was performed according to the Union for International Cancer Control, TNM Classification of Malignant Tumors 7th edition [14].

Patients underwent a geriatric assessment and frailty screening upon their initial consultation at the outpatient clinic. The assessment encompassed multiple domains of health. Physical status was determined by evaluating comorbidities categorized as none, mild, moderate, or severe using the Adult Comorbidity Evaluation (ACE-27) [15, 16]. The intoxication status was determined by considering smoking and drinking behaviors as reported in a standardized health questionnaire. Additionally, the risk of malnutrition was stratified as low, medium, or high based on the Malnutrition Universal Screening Tool (MUST) [17].

Functional status was evaluated through several parameters. Mobility restrictions were determined using the Timed Up & Go test (TUG) [18, 19]. Limitations in activities of daily living were assessed using both the Katz Index of Activities of Daily Living (K-ADL) [20] and the Lawton Index of Instrumental Activities of Daily Living (L-IADL) [21, 22]. Furthermore, patients’ history of falls was taken into account.

Psychological status was evaluated by considering cognitive function, as measured by the Mini Mental State Examination (MMSE) [23, 24]. The risk of depression was assessed using the Geriatric Depression Scale (GDS-15) [25, 26]. Additionally, the potential for delirium was evaluated in accordance with the VMS security program for Dutch hospitals [27].

Frailty status was assessed by the Groningen Frailty Indicator (GFI) and the Geriatric-8 screening tool (G8), chosen for their established validity and reliability in assessing frailty across diverse population. The GFI, a 15-item questionnaire, evaluates physical, cognitive, and social domains of frailty of the older population [28]. The G8, comprises eight items, specifically targets vulnerabilities in older oncological patients, focusing on domains such as nutrition, physical function, and comorbidities [29].

The questionnaires were completed by the patient at the outpatient clinic together with a researcher or a nurse, or completed later at home and returned by mail. Cut-off scores were used to identify patients at declined or at-risk status. The specific cut-off values that were used in this study are presented in Supplementary Table 1.

### Outcome Measures

Swallowing-related quality of life was evaluated using the Head and Neck Swallowing domain (HNSW-QoL) of the European Organization for Research and Treatment of Cancer Quality of Life Questionnaire, Head and Neck cancer module (EORTC QLQ-H&N35) [30, 31]. The HNSW-QoL domain comprises a set of four questions designed to identify the decline in quality of life related to the ability to swallow liquids, pureed food, and solid food, as well as the occurrence of choking events. Responses were assessed using a four-point Likert scale, where higher scores corresponded to greater difficulties. Following the guidelines of the test, a linear transformation was applied to the raw total score, to standardize the scale and present the total score of the swallowing domain uniformly within a range from 0 to 100 [32]. This standardized representation facilitates the interpretation and comparison of results.

Dysphagia, as an adverse event, was characterized by a toxicity grade according to the Common Terminology Criteria for Adverse Events version 4.0 (CTCAE) [33]. The classification approach was derived from the dysphagia scale of CTCAE (CTCAE-D), implemented in previous studies [34, 35]. At UMCG, the CTCAE-D scores were consistently evaluated by a radiation oncologist because of the expertise in the field and to ensure standardized assessment across all patients. The CTCAE-D include questions taking into account the swallowing performance, oral intake adjustments, and health-related consequences due to cancer treatment toxicity. The UMCG toxicity grade score ranges from 0 to 5, each score indicating varying levels of swallowing impairment and dysphagia severity: from normal swallowing function (grade 0), through regular diet (grade 1), altered diet (grade 2), severe symptoms limiting oral intake (grade 3), life-threatening dysphagia (grade 4) to death related to dysphagia (grade 5) (Supplementary Table 1).

Data for HNSW-QoL and CTCAE-D were collected at baseline, defined as the assessment before the initiation of treatment for primary or recurrent HNC, and during subsequent follow-up time points; 3, 6, 12, and 24 months after the completion of treatment.

### Statistical Analyses

Descriptive statistics were used to describe the study population using Statistical Package for the Social Sciences (SPSS) statistics software version 28.0 (IBM Corp., Armonk, New York). Categorical variables were presented as frequencies with corresponding percentages, and continuous variables as mean scores with standard deviations (SDs). Patient characteristics and geriatric assessment scores were defined as potential predictive factors, and HNSW-QoL and CTCAE-D as primary endpoints.

Linear mixed-effect models (LMMs) were employed for the analysis of repeated measures of mean HNSW-QoL scores and CTCAE-D grades, using MLwiN software version 2.02 (Centre for Multilevel Modelling, University of Bristol, United Kingdom). LMM analyses were selected to account for the hierarchical structure of the data, considering the nested nature of observations within each individual and across multiple time points (baseline, 3, 6, 12, and 24 months). This methodology accommodates both the individual-specific variations and the temporal dependencies inherent in the dataset, thereby, offering a robust approach to analyzing complex longitudinal data [36, 37].

The initial step involved a univariable linear regression analysis adjusted for baseline dysphagia scores to account for initial conditions. Variables showing statistical significance at *p*-value threshold of 0.1 in the univariable analysis were considered for inclusion in the subsequent multivariate models. Secondly, a forward stepwise regression analysis was performed to systematically construct a multivariable statistical model. Starting with an empty model, variables were sequentially added based on their contribution to explaining the observed outcomes. The iterative process continued until no additional variables significantly enhanced the model. The adjusted R-squared value was considered to assess the goodness of fit for the selected model.

## Results

### Patient, Tumor, and Treatment Characteristics

In this study, a total of 369 patients were included, and 242 remained eligible for analysis after applying the exclusion criteria (Supplementary Fig. 1). The mean age of the study population was 65.5 years (SD 10.5), and the majority was male (66.1%). Patients were predominantly in a relationship (63.6%), and were living independently (78.5%). The most prevalent tumor localizations were the lip and oral cavity (33.9%), and the larynx (33.1%). Squamous cell carcinoma was the predominant histological type of cancer encountered (90.1%), with a substantial proportion of cases diagnosed at an advanced stage, specifically stage IV (45.5%). As primary treatment modality, most patients underwent surgery (32.2%). Other treatment modalities were radiotherapy (31%), chemoradiotherapy (19%) or a combination of treatment modalities, such as surgery with postoperative radiotherapy (16.1%), and surgery with postoperative chemoradiotherapy (1.2%) (Table 1).

**Table 1:** Patient, tumor, and treatment characteristics

Domain	Variables	Characteristics	Result (*n*, %)
Patient characteristics	Age	Mean SD Median *≥* 65 years < 65 years	65.5 (*±* 10.55) 65 123 (50.8) 119 (49.2)
	Sex	Male Female	160 (66.1) 82 (33.9)
	Marital status	In a relationship Single *Missing*	154 (63.6) 62 (25.6) *26 (10.7)*
	Living situation	Independent Assisted Nursing home *Missing*	190 (78.5) 22 (9.1) 2 (0.8) *28 (11.6)*
Tumor characteristics	Reason for referral	1st primary tumor 2nd, 3rd and 4th primary tumor Recurrent tumor *Missing*	218 (90.1) 13 (5.4) 9 (3.7) *2 (0.8)*
	Location	Lip and oral cavity Larynx Oropharynx Hypopharynx Unknown primary tumor Nasopharynx	82 (33.9) 80 (33.1) 59 (24.4) 9 (3.7) 8 (3.3) 4 (1.7)
	Histopathology	Squamous Cell Carcinoma Other *Missing*	218 (90.1) 13 (5.4) *11 (4.5)*
	Stage	I II III IV *Missing*	62 (25.6) 36 (14.9) 32 (13.2) 110 (45.5) *2 (0.8)*
Treatment characteristics	Primary treatment modality	Surgery Surgery only Post-operative radiotherapy Post-operative chemo-radiotherapy Re-excision Immunotherapy Radiotherapy Salvage surgery Re-irradiation Chemo-radiotherapy Salvage surgery Re-irradiation	121 (50) 47 (19.4) 39 (16.1) 3 (1.2) 31 (12.8) 1 (0.4) 75 (31) 3 (1.2) 3 (1.2) 46 (19) 3 (1.2) 2 (0.8)

### Geriatric Assessment

Regarding physical status, a substantial proportion of participants exhibited moderate to severe comorbidities (38.8%), were current or previous smokers (84.7%) and currently or previously alcohol consumers (74.4%). Nutritionally, a notable subset of patients presented with a medium to high risk of malnutrition (24.8%). A high proportion of patients had restrictions in L-IADL (59.1%), but not in K-ADL (7.9%). Lastly, a substantial higher proportion of patients were classified as frail when using the G8 (55%), in comparison to the GFI (27.3%) (Table 2).

**Table 2:** Geriatric assessment data of the cohort

Domain	Variable	Assessment Tool	Characteristics	Result (*n*, %)
Physical status	Comorbidity	ACE 27	None / mild Moderate / severe	148 (61.2) 94 (38.8)
	Intoxication	Current smoker or history of smoking	No Yes *Missing*	28 (11.6) 205 (84.7) *9 (3.7)*
		Active alcohol consumption or history of alcohol consumption	No Yes *Missing*	51 (21.1) 180 (74.4) *11 (4.5)*
	Nutrition	MUST	Low risk Medium risk High risk *Missing*	166 (68.6) 21 (8.7) 39 (16.1) *16 (6.6)*
Functional status	Mobility	TUG	No restrictions Declined mobility *Missing*	201 (83.1) 27 (11.2) *14 (5.8)*
	Activities of Daily Living	Katz ADL	No restrictions Restrictions *Missing*	197 (81.4) 19 (7.9) *26 (10.7)*
	Instrumental Activities of Daily Living	Lawton IADL	No restrictions Restrictions *Missing*	98 (40.5) 143 (59.1) *1 (0.4)*
	Fall risk	Fall risk	No risk Risk *Missing*	195 (80.6) 15 (6.2) *32 (13.2)*
Psychologic status	Cognition	MMSE	Normal cognitive function Declined cognitive function *Missing*	212 (87.6) 28 (11.6) *2 (0.8)*
	Depression	GDS-15	No depression Depression *Missing*	193 (79.8) 20 (8.3) *29 (12)*
	Delirium risk	Delirium risk	No risk Risk *Missing*	135 (55.8) 104 (43) *3 (1.2)*
Frailty	Frailty screening	GFI	Non-frail Frail *Missing*	150 (62) 66 (27.3) *26 (10.7)*
		G8	Non-frail Frail *Missing*	105 (43.4) 133 (55) *4 (1.7)*

Legend: ACE = Adult Comorbidity Evaluation 27; MUST = Malnutrition Universal Screening Tool; TUG = Timed Up & Go; Katz-ADL = Katz Index of Activities of Daily Living; Lawton-IADL = Lawton Index of Instrumental Activities of Daily Living; MMSE = Mini Mental State Examination; GDS-15 = Geriatric Depression Scale; GFI = Groningen Frailty Indicator; G8 = Geriatric Screening Tool

### Association Between Frailty Status and Swallowing-Related Quality of Life

The evaluation of HNSW-QoL conducted on the cohort revealed fluctuations over time following treatment. Initially, the total score showed a baseline mean of 14.96 (SD 23.23), which increased to 27.38 (SD 25.59) after 3 months, indicating a deterioration in HNSW-QoL. This was followed by a continuous decline, culminating in a score of 12.38 (SD 16.25) at 24 months post-treatment. A similar pattern was observed across the individual questions, such as drinking, eating pureed food, eating solid food, and complications related to choking. Of particular note was the peak of symptoms reported at 3 months, particularly concerning eating solid food (mean 2.21, SD 1.18) (Table 3).

**Table 3:** Outcome measures

Variable	Results				
	Baseline	Month			
		3	6	12	24
HNSW-QoL (mean score, SD)					
Total score *Missing* Drinking Eating pureed food Eating solid food Complications choking	14.96 (23.23) (*n* = 214) *28 (11.6)* 1.54 (0.88) (*n* = 214) 1.36 (0.75) (*n* = 210) 1.67 (1.01) (*n* = 212) 1.22 (0.58) (*n* = 213)	27.38 (25.59) (*n* = 136) *106 (43.8)* 1.84 (1.00) (*n* = 132) 1.75 (1.01) (*n* = 131) 2.21 (1.18) (*n* = 130) 1.48 (0.69) (*n* = 137)	18.39 (22.02) (*n* = 116) *126 (52.1)* 1.46 (0.74) (*n* = 114) 1.43 (0.82) (*n* = 115) 1.86 (1.05) (*n* = 115) 1.39 (0.64) (*n* = 113)	15.91 (20.55) (*n* = 100) *142 (58.7)* 1.44 (0.75) (*n* = 100) 1.36 (0.77) (*n* = 100) 1.75 (0.98) (*n* = 99) 1.33 (0.62) (*n* = 99)	12.38 (16.25) (*n* = 85) *157 (64.9)* 1.37 (0.57) (*n* = 85) 1.15 (0.50) (*n* = 83) 1.58 (0.83) (*n* = 84) 1.35 (0.64) (*n* = 85)
CTCAE-D					
Total score (mean, SD)	0.96 (1.10) (*n* = 171)	1.56 (1.23) (*n* = 165)	1.03 (1.04) (*n* = 138)	0.90 (0.97) (*n* = 123)	0.67 (0.87) (*n* = 96)
*Missing*	*71 (29.3)*	*77 (31.8)*	*104 (43)*	*119 (49.2)*	*146 (60.3)*
Grades (n, %)					
0 I II III IV V	86 (35.5) 26 (10.7) 38 (15.7) 21 (8.7) 0 0	48 (19.8) 33 (13.6) 27 (11.2) 57 (23.6) 0 0	55 (22.7) 41 (16.9) 24 (9.9) 18 (7.4) 0 0	54 (22.3) 37 (15.3) 22 (9.1) 10 (4.1) 0 0	53 (21.9) 25 (10.3) 14 (5.8) 4 (1.7) 0 0

Legend: HNSW-QoL = EORTC QLQ – H&N35 Swallowing domain; CTCAE-D = Common Terminology Criteria for Adverse Events – Dysphagia

Interpretation: Higher scores means higher level of symptoms, i.e. more swallowing-related quality of life complaints, and more toxicity-related swallowing complaints

We conducted univariable LMM analyses, adjusted for baseline HNSW-QoL. Several variables had significant associations: mobility (TUG) (β = 9.973, *p*-value = 0.055), fall risk (β = 19.854, *p*-value = 0.054), depression (GDS-15) (β = 10.024, *p*-value = 0.040), and frailty status measured by GFI (β = 10.936, *p*-value = 0.001) and G8 (β = 11.770, *p*-value < 0.001) (Table 4).

**Table 4:** Univariable linear mixed model analysis of patient characteristics and geriatric assessment for predicting post-treatment swallowing-related quality of life measured by HNSW-QoL

Variable	Reference	Univariable adjusted by HNSW-QoL at baseline		
		Estimate (β)	S.E.	*p*-value
Age	< 65 y	3.343	3.079	0.278
Age	Continuous	0	0.160	1
Sex	Male	3.975	3.424	0.246
Marital status	Single	-3.977	3.427	0.246
Living situation	Independent	0.101	6.800	0.988
ACE-27	None/mild	3.290	3.206	0.305
History of smoking	No	4.663	4.897	0.341
History of drinking	No	-5.610	3.976	0.158
MUST	Low risk	2.868	2.174	0.187
**TUG**	**No restrictions**	**9.973**	**5.194**	**0.055**
Katz-ADL	No restrictions	-2.618	7.302	0.720
Lawton-IADL	No restrictions	0.666	3.109	0.830
**Fall risk**	**No**	**19.854**	**10.302**	**0.054**
MMSE	Normal function	8.491	6.217	0.172
**GDS-15**	**No**	**10.024**	**4.880**	**0.040**
Delirium risk	No	-0.677	3.122	0.828
**GFI**	**Non-frail**	**10.936**	**3.327**	**0.001**
**G8**	**Non-frail**	**11.770**	**3.060**	**< 0.001**

Legend: ACE = Adult Comorbidity Evaluation 27; MUST = Malnutrition Universal Screening Tool; TUG = Timed Up & Go; Katz-ADL = Katz Index of Activities of Daily Living; Lawton-IADL = Lawton Index of Instrumental Activities of Daily Living; MMSE = Mini Mental State Examination; GDS-15 = Geriatric Depression Scale; GFI = Groningen Frailty Indicator; G8 = Geriatric Screening Tool. All variables are dichotomous except for age. Significant p-values values are indicated in bold

Baseline HNSW-QoL consistently exhibited a positive impact, evidenced by consistently low *p*-values in all models (< 0.1). When incorporating frailty status into the model, assessed by either G8 or GFI, both emerged as significant positive predictors (G8: β = 11.770, *p*-value < 0.001; GFI: β = 10.936, *p*-value = 0.001). The subsequent inclusion of additional variables to G8 or GFI, did not yield significant impacts, like GDS-15 (β = 6.840, *p*-value = 0.152; β = 4.121, *p*-value = 0.437), fall risk (β = 14.880, *p*-value = 0.136; β = 14.842, *p*-value = 0.144), and TUG (β = 5.548, *p*-value = 0.281; β = 7.515, *p*-value = 0.141), respectively. For the G8 model, the adjusted R-squared was 0.247, while for the GFI model, it was 0.211.

**Fig. 1:**
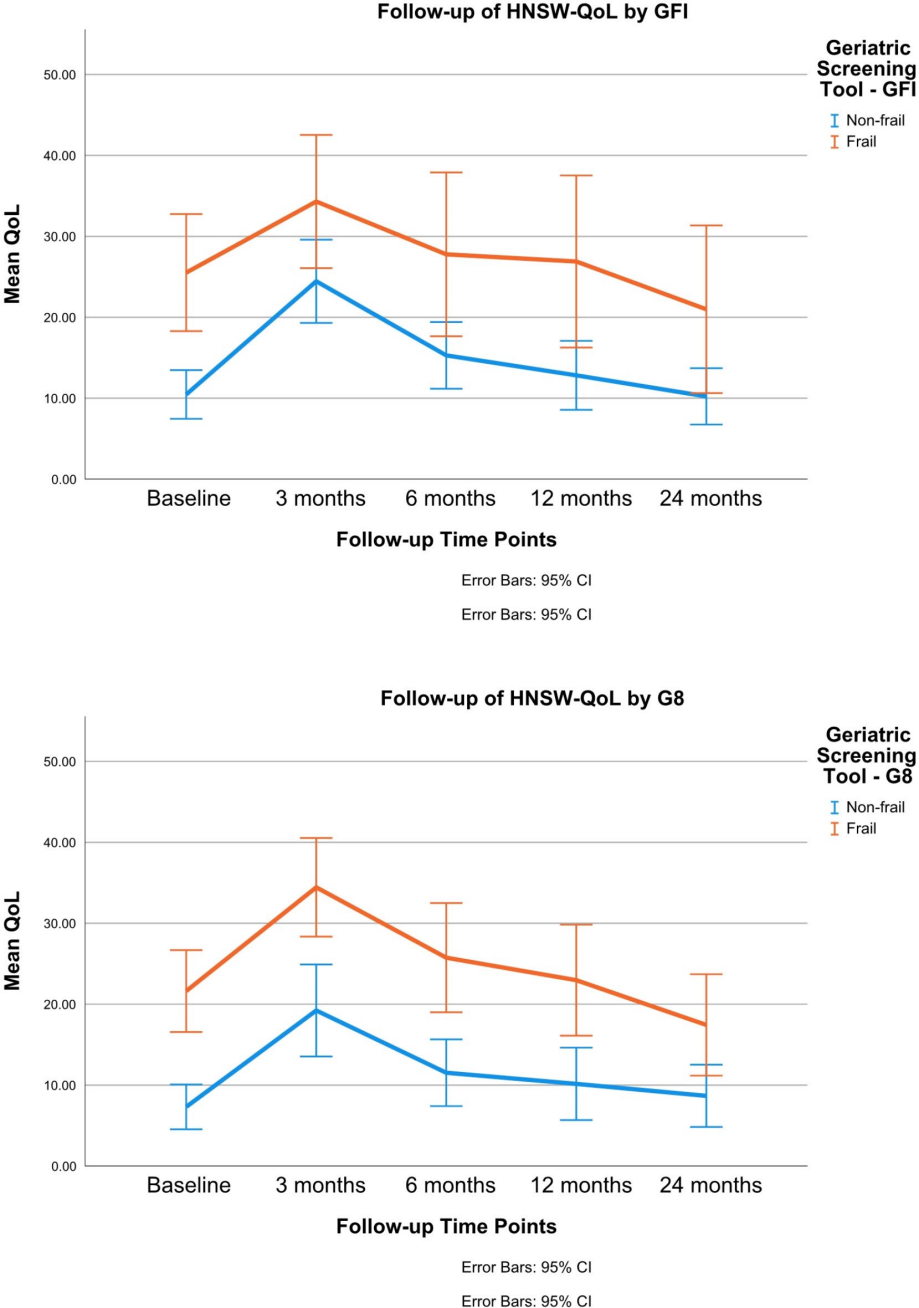
Influence of frailty status on swallowing-related quality of life over time. Legend: HNSW-QoL = EORTC QLQ - H&N35 Swallowing domain; GFI = Groningen Frailty Indicator; G8 = Geriatric Screening Tool. Higher scores means more impaired swallowing-related quality of life

Frail patients, as assessed by GFI or G8, consistently showed worse HNSW-QoL compared to non-frail patients across time points, as evidenced by higher scores. At baseline, frail patients had notably higher HNSW-QoL scores than non-frail patients. At 3 months post-treatment, both groups had a deterioration in HNSW-QoL. This disparity persisted up to 24 months post-treatment, with both groups showing gradual improvement over time (Fig. 1).

### Association Between Frailty Status and Toxicity-Related Dysphagia

Fluctuations in CTCAE-D assessments were also observed at all time points, with the mean score starting at 0.96 (SD 1.10) at baseline, increasing to 1.56 (SD 1.23) at 3 months, and followed by a gradual decrease to 0.67 (SD 0.87) at 24 months post-treatment (Table 3).

In univariable LMM analysis, adjusted for baseline CTCAE-D, several variables had significant associations with CTCAE-D, including fall risk (β = 0.580, *p*-value = 0.077), cognition (MMSE) (β = 0.454, *p*-value = 0.042), and frailty status assessed by GFI (β = 0.331, *p*-value = 0.019), and G8 (β = 0.245, *p*-value = 0.058) (Table 5).

**Table 5:** Univariable linear mixed model analysis of patient characteristics and geriatric assessment for predicting post-treatment dysphagia measured by CTCAE-D

Variable	Reference	Univariable analysis adjusted by CTCAE-D at baseline		
		Estimate (β)	S.E.	*p*-value
Age	< 65 y	0.134	0.122	0.272
Age	Continuous	-0.003	0.006	0.617
Sex	Male	0.121	0.138	0.381
Marital status	Single	-0.170	0.143	0.235
Living situation	Independent	0.305	0.255	0.232
ACE-27	None/mild	0.083	0.128	0.517
History of smoking	No	0.022	0.194	0.910
History of drinking	No	0.179	0.163	0.272
MUST	Low risk	0.128	0.081	0.114
TUG	No restrictions	0.309	0.212	0.145
Katz-ADL	No restrictions	-0.342	0.281	0.224
Lawton-IADL	No restrictions	0.069	0.123	0.575
**Fall risk**	**No**	**0.580**	**0.328**	**0.077**
**MMSE**	**Normal function**	**0.454**	**0.233**	**0.042**
GDS-15	No	0.255	0.208	0.220
Delirium risk	No	0.025	0.123	0.839
**GFI**	**Non-frail**	**0.331**	**0.141**	**0.019**
**G8**	**Non-frail**	**0.245**	**0.129**	**0.058**

Legend: ACE = Adult Comorbidity Evaluation 27; MUST = Malnutrition Universal Screening Tool; TUG = Timed Up & Go; Katz-ADL = Katz Index of Activities of Daily Living; Lawton-IADL = Lawton Index of Instrumental Activities of Daily Living; MMSE = Mini Mental State Examination; GDS-15 = Geriatric Depression Scale; GFI = Groningen Frailty Indicator; G8 = Geriatric Screening Tool. All variables are dichotomous except for age. Significant p-values values are indicated in bold

Through stepwise forward analysis, the presence of CTCAE-D at baseline also consistently demonstrated a significant positive effect across all models (*p*-value < 0.1). The optimal model for predicting CTCAE-D included baseline CTCAE-D as a covariate, along with frailty status assessed by either G8 (β = 0.245, *p*-value = 0.058), or GFI (β = 0.331, *p*-value = 0.019) as significant positive predictors. Adding variables to the GFI model, such as MMSE (β = 0.294, *p*-value = 0.228), and fall risk (β = 0.422, *p*-value = 0.190), did not result in a statistically significant improvement of the predictive model. In the G8 model, the inclusion of MMSE (β = 0.392, *p*-value = 0.081), and fall risk (β = 0.506, *p*-value = 0.126), rendered G8 not significant (β = 0.204, *p*-value = 0.114; β = 0.179, *p*-value = 0.195, respectively). The adjusted R-squared for the GFI model was 0.443, and for the G8 model 0.445.

**Fig. 2:**
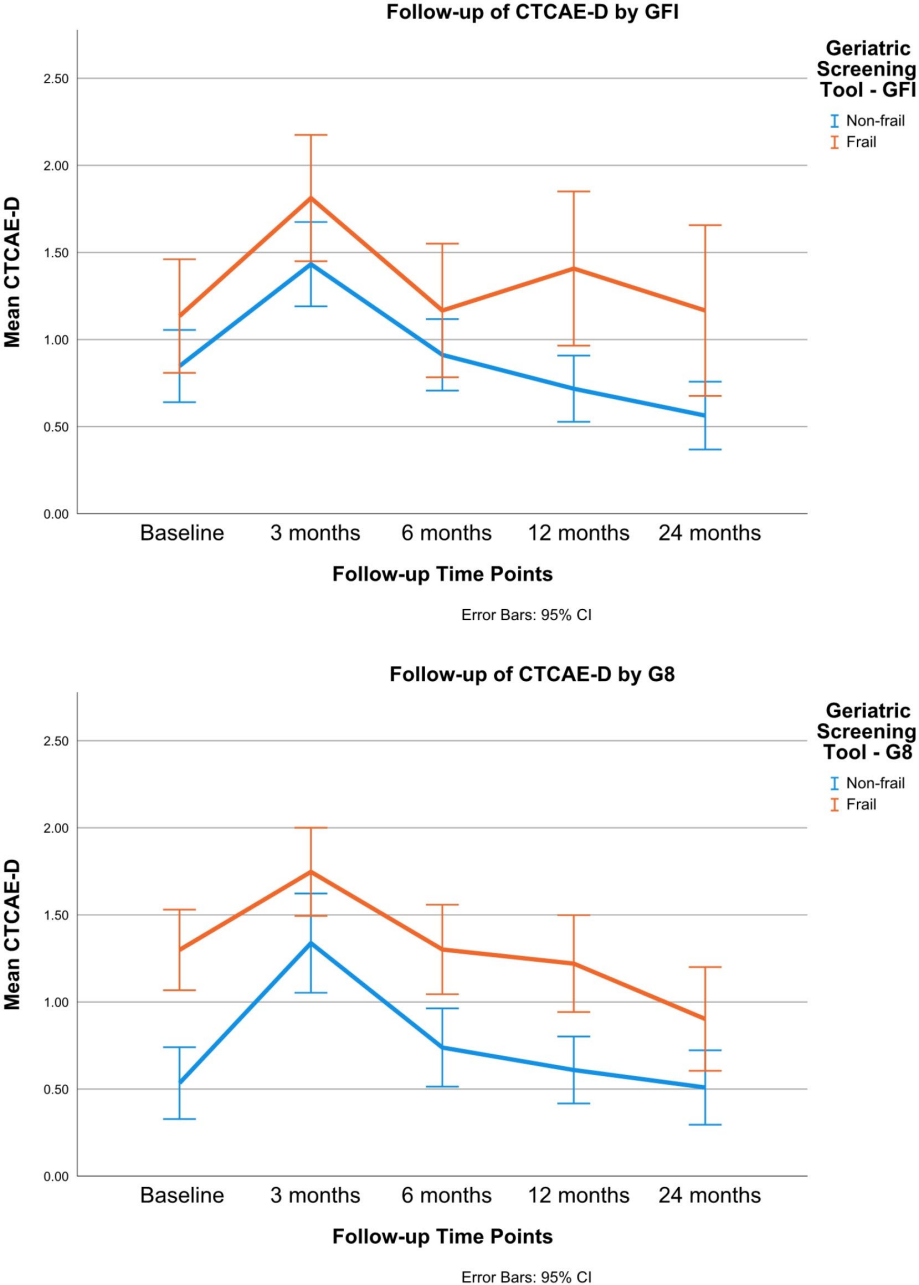
Influence of frailty status on toxicity-related dysphagia over time. Legend: CTCAE-D = Common Terminology Criteria for Adverse Events - Dysphagia; GFI = Groningen Frailty Indicator; G8 = Geriatric Screening Tool. Higher scores means higher toxicity-related swallowing problems

Frail patients, as assessed by GFI or G8, consistently had higher CTCAE-D scores than non-frail patients across time points. At baseline, frail patients had higher CTCAE-D scores compared to non-frail patients. While both groups experienced fluctuations, frail patients consistently had higher scores throughout the follow-up period (Fig. 2).

## Discussion

This study investigated the relationship between pre-treatment frailty status and post-treatment dysphagia in patients with head and neck cancer (HNC). Frail patients exhibited a poorer course of HNSW-QoL and CTCAE-D before treatment and at all time-points up to 24 months after treatment. There was a statistically significant association between frailty, as assessed by the G8 or GFI, and swallowing-related quality of life (HNSW-QoL), and a statistically significant association between G8 or GFI, and toxicity-related dysphagia (CTCAE-D). However, it is crucial to note that both frail and non-frail patients experienced significant deterioration in dysphagia outcomes, particularly between baseline and 3 months post-treatment. This suggests that while frailty status is associated with worse overall outcomes, the severity of treatment-related dysphagia impacts a broad spectrum of patients regardless of frailty.

The clinical importance of these findings lies in highlighting the role of frailty, identified through geriatric assessments, in adapting treatment plans for patients with HNC. By integrating frailty screening as a standard pre-treatment assessment, clinicians gain valuable information that allows them to identify at-risk patients, anticipate, and address post-treatment swallowing problems.

Digging into the nuances of frailty and the mechanisms behind our findings reveals a complex and synergistic interactions between various health conditions, including cancer, frailty, malnutrition, and dysphagia. Up to 60% of patients with HNC are malnourished at the time of diagnosis [38–41]. In this study, 24.8% of patients presented with a medium to high risk of malnutrition scored using the MUST. As mentioned previously, frailty and malnutrition may co-exist. Furthermore, swallowing problems are related to malnutrition [38]. Dysphagia is one of the most common side effects during and after treatment and represents one of the main causes of malnutrition in patients with HNC. Patients who initially were not malnourished, may experience a gradual worsening of nutritional status because of treatment toxicity. Other factors that play a part in putting patients at nutritional risk are metabolic and inflammatory abnormalities related to cancer, which can cause hypermetabolism and inadequate absorption of nutrients [42]. Additionally, the location of the tumor can directly influence chewing and swallowing ability as well as food tolerance [43, 44]. In a previous study by our group, almost 20% of patients were frail and presented with a high risk of malnutrition [45]. Therefore, the interaction between malnutrition, frailty, dysphagia, and other treatment-related factors can lead to a complex health condition.

Regarding frailty, we used two screening tools in this study. When using the G8, 55% of patients were classified as frail versus 27.3% of patients when using the GFI. Baitar et al. evaluated the diagnostic performance of both screening tools against the CGA. Both the G8 and GFI were able to separate patients with cancer with a normal and abnormal CGA [46]. This study was performed in a population of patients with various types and stages of cancer. The outcomes may be different in patients with HNC, because our patients often present with feeding difficulties. Three out of eight questions of the G8 are feeding-related, whereas only one out of 15 questions of the GFI is feeding-related. This explains why in the present study 55% were considered frail when using the G8 and only 27.3% when using the GFI. However, these differences in the frailty screening tools did not influence the outcome, as both the patient and the physician rated dysphagia scores were significantly higher in the frail groups, regardless of the frailty screener. For oncological studies, G8 is more often used; however, it also has its obvious drawbacks, like several questions related to the malnutrition which is more often altered in HNC patients. Our findings suggest that G8 has higher sensitivity, capturing a broader range of patients at risk; however, presumably the specificity is lower. If a choice has to be made which frailty screener should be used, we suggest G8, as with this instrument less frail patients would miss the comprehensive geriatric assessment. However, having more patients failed the screening, the workload of the geriatrician would increase.

Previous studies have shown significant differences in general health-related QoL between frail and non-frail patients [47, 48]. This was also true for specific domains of health-related QoL. These studies did not analyze swallowing-related QoL specifically. No studies have been published evaluating the association between pre-treatment frailty and post-treatment swallowing-related QoL. Regarding clinician-rated toxicity, an earlier study did not find an association between pre-treatment geriatric disabilities and treatment-related toxicity, including dysphagia, in patients with HNC [35]. In contrast, in our study, frail patients (defined by G8 or GFI before treatment) had higher CTCAE-D during the whole follow-up period. The differing outcomes can be explained by their different setups. First, the cited study focused only on acute radiation-induced toxicity, with weekly measurements during the first 7 weeks of treatment and a last measurement at 12 weeks after treatment initiation. In contrast, in the present study we evaluated outcomes between 3 and 24 months after treatment completion. As curative radiation therapy of HNC typically involves 35 fractions in 7 weeks, the 12 week measurement in the study of 2022 was taken 5 weeks after finishing treatment. In that study, dysphagia toxicity scores were significantly altered 3 weeks after starting radiation therapy and remained affected throughout the whole 12 week follow-up period. Secondly, the cited study used a combined toxicity score from 10 CTCAE scales, including dysphagia, whereas our study focused specifically on the toxicity-related dysphagia scale. The cited study observed slight overall differences in radiation toxicity scores between frail and non-frail patients at baseline, likely due to the tumor itself rather than radiation therapy; however, over time, the increase in toxicity for frail patients was not greater than for non-frail patients.

The available literature on the relationship between frailty and dysphagia in patients with cancers in other locations than the head and neck is also limited. Dysphagia occurs less frequently in patients with solid malignancies outside the head, neck, and upper gastrointestinal tract with a prevalence of 19% [49]. Dysphagia in these patients has been shown to be associated with functional decline, cachexia, and increased weight loss, all of which are considered symptoms of or associated with frailty [49]. In a study evaluating patients who underwent thyroid or parathyroid surgery for benign or malignant disease, frailty was associated with preoperative swallowing dysfunction [50]. Although preoperative swallowing dysfunction was associated with an increased likelihood of postoperative dysphagia, frailty was not associated with postoperative dysphagia. Sahli et al. did not find a significant relation between frailty and swallowing changes following thyroidectomy for benign or malignant disease [51]. In contrast to our findings, a study in patients with esophageal malignancies also did not show a relation between frailty status and postoperative dysphagia [52]. These contrasting findings may be explained by the differences in the cohorts (other primary site, other treatment regimens) or by the different screening tools. Frailty was measured in these studies using other instruments than we used in our study, namely the Fried Frailty Index, the modified Frailty Index and the Johns Hopkins Adjusted Clinical Groups frailty-defining diagnosis indicator. Dysphagia was measured using the Dysphagia Handicap Index questionnaire and patient-reported swallowing changes and dysphagia.

Our study has several clinical implications. First, the integration of frailty assessment into routine pre-treatment evaluations emerges as a critical step in enhancing personalized treatment strategies for the older population with HNC. Second, the identified associations between frailty and dysphagia underscore the potential for proactive interventions aimed at improving post-treatment outcomes. Clinicians can implement targeted interventions to mitigate the impact of dysphagia. Lastly, it is known that head and neck cancer patients are frailer than patients with other solid malignancies [5]; therefore, applying a strict age threshold for studies dealing with geriatric deficits of head and neck cancer patients would not provide the correct data. Our study’s emphasis on “geriatric deficits” rather than focusing solely on an older cohort reflects the broader utility of geriatric assessment tools beyond the traditional age threshold of 65 years. Although patients older than 65 years are often classified as elderly, the concept of frailty and the use of comprehensive geriatric assessments (CGAs) are relevant to younger patients with HNC who share similar vulnerabilities. This inclusive approach allows us to encompass a diverse population of patients who may biologically exhibit accelerated aging processes, thus enhancing our understanding of the complex interplay between frailty and post-treatment dysphagia. By including patients of different ages who exhibit geriatric-like deficits, we aim to thoroughly evaluate how frailty affects treatment outcomes, offering insights that might be overlooked if our study were limited to those over 65 years of age.

While this study provides valuable insights, it is essential to acknowledge certain limitations. The absence of information regarding treatments by a speech and language pathologist focused on swallowing problems, and the influence of nutritional management by dietitians during HNC treatment warrants attention, as these factors could interfere with the observed outcomes. Additionally, the high percentage of missing data, with 43.8% of HNSW-QoL data and 31.8% of CTCAE-D data missing at the first follow-up (3 months) and continuous increase, could impact the interpretation and generalizability of our findings. Missing data is common in data-biobanks, especially when there are many variables and outcome measures across multiple time-points. Complete case analysis often results in important bias in such cohorts, as the data is typically not missing at random [53]. Despite these limitations, the study’s strengths lie in the prospective inclusion and use of a comprehensive database, providing a robust foundation for our analyses. The statistical analyses employed is an optimal model for leading against missing data, further enhance the robustness of the findings, as it allows for analysis without discarding entire cases and maximizes data usage. Additionally, the use of internationally validated geriatric assessments and outcome measures, ensures the reliability and relevance of our findings.

For future perspectives, it is important to validate these findings in larger multicenter cohorts to enhance generalizability. Additionally, exploring the efficacy of targeted interventions, such as speech therapy and nutritional support tailored for frail patients, is crucial. Given the complex nature of frailty in HNC patients, developing comprehensive care protocols that promotes synergistic treatment strategies could improve both short- and long-term outcomes.

## Conclusions

As the older and vulnerable population facing head and neck cancer (HNC) increases, geriatric assessment has emerged as a vital tool to guide personalized treatment strategies. This study highlights the impact of frailty on both swallowing-related quality of life and toxicity-related dysphagia over time. Frailty screened with GFI or G8 was consistently associated with worse swallowing-related quality of life and higher toxicity-related dysphagia. However, both frail and non-frail patients experienced significant declines in dysphagia outcomes, particularly within the first 3 months post-treatment. These findings may be used as relevant pre-treatment information to identify patients with HNC who are likely to face swallowing issues because of intensive treatment. Therefore, the need to integrate frailty screening into pre-treatment assessments is imperative, as this information may help in the decision making and may allow interventions to optimize post-treatment swallowing outcomes.

## Electronic Supplementary Material

Below is the link to the electronic supplementary material.

Supplementary Material 1

## Data Availability

The dataset generated and analyzed during the current study is available from the corresponding author upon reasonable request.

## References

[1] Kinsella K, Phillips DR. Global aging: the challenge of success. Popul Bull. 2005;60(1).

[2] Bray F, Ferlay J, Soerjomataram I, Siegel RL, Torre LA, Jemal A. Global cancer statistics 2018: GLOBOCAN estimates of incidence and mortality worldwide for 36 cancers in 185 countries. Cancer J Clin. 2018;68(6):394–424. 10.3322/caac.21492 30207593

[3] Pezzuto F, Buonaguro L, Caponigro F, Ionna F, Starita N, Annunziata C, et al. Update on Head and Neck Cancer: current knowledge on epidemiology, risk factors, molecular features and Novel therapies. Oncology. 2015;89(3):125–36. 25967534 10.1159/000381717

[4] van Deudekom FJ, Schimberg AS, Kallenberg MH, Slingerland M, van der Velden LA, Mooijaart SP. Functional and cognitive impairment, social environment, frailty and adverse health outcomes in older patients with head and neck cancer, a systematic review. Oral Oncol. 2017;64:27–36. 28024721 10.1016/j.oraloncology.2016.11.013

[5] Bras L, Driessen DAJJ, de Vries J, Festen S, van der Laan BFAM, van Leeuwen BL, et al. Patients with head and neck cancer: are they frailer than patients with other solid malignancies? Eur J Cancer Care. 2020;29(1):e13170. 10.1111/ecc.13170 PMC7063690 31571340

[6] Clegg A, Young J, Iliffe S, Rikkert MO, Rockwood K. Frailty in elderly people. Lancet. 2013;381(9868):752–62. 23395245 10.1016/S0140-6736(12)62167-9 PMC4098658

[7] Extermann M, Hurria A. Comprehensive Geriatric Assessment for older patients with Cancer. JCO. 2007;25(14):1824–31. 10.1200/JCO.2007.10.6559 17488980

[8] Soto-Perez-de-Celis E, Li D, Yuan Y, Lau YM, Hurria A. Functional versus chronological age: geriatric assessments to guide decision making in older patients with cancer. Lancet Oncol. 2018;19(6):e305–16. 29893262 10.1016/S1470-2045(18)30348-6

[9] Thankappan K, Iyer S, Menon JR. Dysphagia Management in Head and Neck cancers. Springer; 2018.

[10] Govender R, Smith CH, Taylor SA, Barratt H, Gardner B. Swallowing interventions for the treatment of dysphagia after head and neck cancer: a systematic review of behavioural strategies used to promote patient adherence to swallowing exercises. BMC Cancer. 2017;17(1):43. 28068939 10.1186/s12885-016-2990-x PMC5223405

[11] Denaro N, Merlano MC, Russi EG. Dysphagia in Head and Neck Cancer patients: pretreatment evaluation, predictive factors, and Assessment during Radio-Chemotherapy, recommendations. Clin Exp Otorhinolaryngol. 2013;6(3):117. 24069513 10.3342/ceo.2013.6.3.117 PMC3781223

[12] Baijens LW, Clavé P, Cras P, Ekberg O, Forster A, Kolb G, et al. European Society for Swallowing Disorders - European Union Geriatric Medicine Society white paper: oropharyngeal dysphagia as a geriatric syndrome. CIA. 2016;11:1403–28. 10.2147/CIA.S107750 PMC5063605 27785002

[13] Sidorenkov G, Nagel J, Meijer C, Duker JJ, Groen HJM, Halmos GB, et al. The OncoLifeS data-biobank for oncology: a comprehensive repository of clinical data, biological samples, and the patient’s perspective. J Transl Med. 2019;17(1):374. 31727094 10.1186/s12967-019-2122-x PMC6857242

[14] Sobin LH, Gospodarowicz MK, Wittekind C. TNM classification of malignant tumours. 7th ed. Oxford, UK: Wiley-Blackwell; 2009.

[15] Piccirillo JF. Importance of Comorbidity in Head and Neck Cancer. Laryngoscope. 2000;110(4):593–602. 10764003 10.1097/00005537-200004000-00011

[16] Piccirillo JF. Prognostic importance of Comorbidity in a hospital-based Cancer Registry. JAMA. 2004;291(20):2441. 15161894 10.1001/jama.291.20.2441

[17] Boléo-Tomé C, Monteiro-Grillo I, Camilo M, Ravasco P. Validation of the Malnutrition Universal Screening Tool (MUST) in cancer. Br J Nutr. 2012;108(2):343–8. 22142968 10.1017/S000711451100571X

[18] Podsiadlo D, Richardson S. The timed up & go: a test of Basic Functional mobility for Frail Elderly persons. J Am Geriatr Soc. 1991;39(2):142–8. 1991946 10.1111/j.1532-5415.1991.tb01616.x

[19] Barry E, Galvin R, Keogh C, Horgan F, Fahey T. Is the timed up and go test a useful predictor of risk of falls in community dwelling older adults: a systematic review and meta- analysis. BMC Geriatr. 2014;14(1):14. 24484314 10.1186/1471-2318-14-14 PMC3924230

[20] Katz S. Studies of illness in the aged: the Index of ADL: a standardized measure of biological and psychosocial function. JAMA. 1963;185(12):914. 14044222 10.1001/jama.1963.03060120024016

[21] Lawton MP, Brody EM. Assessment of older people: self-maintaining and instrumental activities of daily living. Gerontologist. 1969;9(3):179–86. 5349366

[22] Graf C. The Lawton Instrumental activities of Daily Living Scale. AJN Am J Nurs. 2008;108(4):52. 18367931 10.1097/01.NAJ.0000314810.46029.74

[23] Folstein MF, Folstein SE, McHugh PR. Mini-mental state. J Psychiatr Res. 1975;12(3):189–98. 1202204 10.1016/0022-3956(75)90026-6

[24] van der Cammen TJM, van Harskamp F, Stronks DL, Passchier J, Schudel WJ. Value of the Mini-mental State examination and informants’ data for the detection of dementia in geriatric outpatients. Psychol Rep. 1992;71(3):1003–E1009. 1454906 10.2466/pr0.1992.71.3.1003

[25] Yesavage JA, Sheikh JI. 9/Geriatric Depression Scale (GDS): recent evidence and development of a shorter version. Clin Gerontologist. 1986;5(1–2):165–73.

[26] Brown LM, Schinka JA. Development and initial validation of a 15-item informant version of the geriatric Depression Scale. Int J Geriat Psychiatry. 2005;20(10):911–8. 10.1002/gps.1375 16163741

[27] VMSzorg. Praktijkgids Kwetsbare Ouderen [Internet]. 2009. https://www.vmszorg.nl/wp-content/uploads/2017/11/web_2009.0104_praktijkgids_kwetsbare_ouderen.pdf

[28] Schuurmans H, Steverink N, Lindenberg S, Frieswijk N, Slaets JPJ. Old or Frail: what tells us more? Journals Gerontol Ser A: Biol Sci Med Sci. 2004;59(9):M962–5. 10.1093/gerona/59.9.m962 15472162

[29] Bellera CA, Rainfray M, Mathoulin-Pélissier S, Mertens C, Delva F, Fonck M, et al. Screening older cancer patients: first evaluation of the G-8 geriatric screening tool. Ann Oncol. 2012;23(8):2166–72. 22250183 10.1093/annonc/mdr587

[30] Bjordal K, de Grae A, Fayers PM, Hammerlid E, van Pottelsberghe C, Curran D et al. A 12 country field study of the EORTC QLQ-C30 (version 3.0) and the head and neck cancer specific module (EORTC QLQ-H&N35) in head and neck patients. Eur J Cancer. 2000. 10.1016/s0959-8049(00)00186-6 10974628

[31] Singer S, Arraras JI, Chie WC, Fisher SE, Galalae R, Hammerlid E, et al. Performance of the EORTC questionnaire for the assessment of quality of life in head and neck cancer patients EORTC QLQ-H&N35: a methodological review. Qual Life Res. 2013;22(8):1927–41. 23188134 10.1007/s11136-012-0325-1

[32] Fayers PM, Aaronson N, Bjordal K, Groenvold M, Curran D, Bottomley A. EORTC QLQ-C30 scoring manual. 3rd ed. Brussels: EORTC; 2001.

[33] National Cancer Institute. Common Terminology Criteria for Adverse Events (CTCAE). 2009; https://www.eortc.be/services/doc/ctc/ctcae_4.03_2010-06-14_quickreference_5x7.pdf

[34] van Rijn-Dekker MI, van den Bosch L, van den Hoek JGM, Bijl HP, van Aken ESM, van der Hoorn A, et al. Impact of Sarcopenia on survival and late toxicity in head and neck cancer patients treated with radiotherapy. Radiother Oncol. 2020;147:103–10. 32251949 10.1016/j.radonc.2020.03.014

[35] de Vries J, Poelman A, Sidorenkov G, Festen S, de Bock GH, Langendijk JA, et al. The association of frailty and outcomes of geriatric assessment with acute radiation-induced toxicity in patients with head and neck cancer. Oral Oncol. 2022;130:105933. 35665634 10.1016/j.oraloncology.2022.105933

[36] Shek DTL, Ma CMS. Longitudinal data analyses using Linear mixed models in SPSS: concepts, procedures and illustrations. Sci World J. 2011;11:42–76. 10.1100/tsw.2011.2 PMC5719989 21218263

[37] Singer JD, Willett JB. Applied longitudinal data analysis: Modeling change and event occurrence. New York, NY, US: Oxford University Press; 2003. xx, 644 p. (Applied longitudinal data analysis: Modeling change and event occurrence).

[38] Jager-Wittenaar H, Dijkstra PU, Vissink A, van Oort RP, van der Laan BFAM, Roodenburg JLN. Malnutrition in patients treated for oral or oropharyngeal cancer—prevalence and relationship with oral symptoms: an explorative study. Support Care Cancer. 2011;19(10):1675–83. 20844902 10.1007/s00520-010-1001-z PMC3166597

[39] Jager-Wittenaar H, Dijkstra PU, Vissink A, Langendijk JA, van der Laan BFAM, Pruim J, et al. Changes in nutritional status and dietary intake during and after head and neck cancer treatment. Head Neck. 2011;33(6):863–70. 20737491 10.1002/hed.21546

[40] Alshadwi A, Nadershah M, Carlson ER, Young LS, Burke PA, Daley BJ. Nutritional considerations for Head and Neck Cancer patients: a review of the literature. J Oral Maxillofac Surg. 2013;71(11):1853–60. 23845698 10.1016/j.joms.2013.04.028

[41] Hébuterne X, Lemarié E, Michallet M, de Montreuil CB, Schneider SM, Goldwasser F. Prevalence of Malnutrition and current use of Nutrition support in patients with Cancer. J Parenter Enter Nutr. 2014;38(2):196–204. 10.1177/0148607113502674 24748626

[42] Matteucci S, De Pasquale G, Pastore M, Morenghi E, Pipitone V, Soekeland F, et al. Low-bacterial Diet in Cancer patients: a systematic review. Nutrients. 2023;15(14):3171. 37513590 10.3390/nu15143171 PMC10385845

[43] Hunter M, Kellett J, Toohey K, D’Cunha NM, Isbel S, Naumovski N. Toxicities caused by Head and Neck Cancer treatments and their influence on the development of Malnutrition: review of the literature. Eur J Investig Health Psychol Educ. 2020;10(4):935–49. 34542427 10.3390/ejihpe10040066 PMC8314324

[44] Mello AT, Borges DS, de Lima LP, Pessini J, Kammer PV, Trindade EBSM. Effect of oral nutritional supplements with or without nutritional counselling on mortality, treatment tolerance and quality of life in head-and-neck cancer patients receiving (chemo)radiotherapy: a systematic review and meta-analysis. Br J Nutr. 2021;125(5):530–47. 32594952 10.1017/S0007114520002329

[45] Dewansingh P, Bras L, ter Beek L, Krijnen WP, Roodenburg JLN, van der Schans CP, et al. Malnutrition risk and frailty in head and neck cancer patients: coexistent but distinct conditions. Eur Arch Otorhinolaryngol. 2023;280(4):1893–902. 36484854 10.1007/s00405-022-07728-6 PMC9988738

[46] Baitar A, Van Fraeyenhove F, Vandebroek A, De Droogh E, Galdermans D, Mebis J, et al. Evaluation of the Groningen Frailty Indicator and the G8 questionnaire as screening tools for frailty in older patients with cancer. J Geriatric Oncol. 2013;4(1):32–8. 10.1016/j.jgo.2012.08.001 24071490

[47] de Vries J, Bras L, Sidorenkov G, Festen S, Steenbakkers RJHM, Langendijk JA, et al. Frailty is associated with decline in health-related quality of life of patients treated for head and neck cancer. Oral Oncol. 2020;111:105020. 33045628 10.1016/j.oraloncology.2020.105020

[48] de Vries J, Bras L, Sidorenkov G, Festen S, Steenbakkers RJHM, Langendijk JA, et al. Association of deficits identified by Geriatric Assessment with Deterioration of Health-Related Quality of Life in patients treated for Head and Neck Cancer. JAMA Otolaryngol Head Neck Surg. 2021;147(12):1089–99. 34673914 10.1001/jamaoto.2021.2837 PMC8532038

[49] Kenny C, Regan J, Balding L, Higgins S, O’Leary N, Kelleher F, et al. Dysphagia Prevalence and predictors in Cancers outside the Head, Neck, and Upper Gastrointestinal Tract. J Pain Symptom Manag. 2019;58(6):949–e9582. 10.1016/j.jpainsymman.2019.06.030 31445137

[50] Crepeau PK, Sutton W, Sahli Z, Fedorova T, Russell JO, Zeiger MA, et al. Prevalence and risk factors for dysphagia in older adults after thyroid and parathyroid surgery. Surgery. 2024;175(1):99–106. 37945476 10.1016/j.surg.2023.04.066 PMC10841879

[51] Sahli Z, Canner JK, Najjar O, Schneider EB, Prescott JD, Russell JO, et al. Association between Age and patient-reported changes in Voice and swallowing after Thyroidectomy. Laryngoscope. 2019;129(2):519–24. 30194684 10.1002/lary.27297 PMC6344315

[52] Shahrestani S, Sayed S, Nasrollahi T, Nasrollahi T, Huang L, McGillivray E, et al. Association between frailty status and complications in patients undergoing surgical excision of malignant esophageal neoplasms. Ann Gastroenterol. 2023;36(5):517–23. 37664228 10.20524/aog.2023.0825 PMC10433248

[53] de Vries J, Vermue DJ, Sidorenkov G, Festen S, Langendijk JA, de Bock GH, et al. Head and neck cancer patients with geriatric deficits are more often non-responders and lost from follow-up in quality of life studies. Eur Arch Otorhinolaryngol. 2024;281(5):2619–26. 38427043 10.1007/s00405-024-08528-w PMC11024024

