# Supplementary material for: Impact of Frailty on Post-Treatment Dysphagia in Patients with Head and Neck Cancer
Source: Dysphagia. 2024 Aug 28;40(3):563–75. doi: 10.1007/s00455-024-10754-7 (PMC12145293; doi:10.1007/s00455-024-10754-7)
Supplement: Supplementary file 1 — Supplementary Material 1 [file 455_2024_10754_MOESM1_ESM.pdf]

Hurtado-Oliva J., van der Laan H.P., de Vries J., Steenbakkers R.J.H.M., Halmos G.B., Wegner I (2024). Impact of Frailty on Post-Treatment Dysphagia in Patients with Head and Neck Cancer, Journal of Dysphagia, doi: https://doi.org/10.1007/s00455-024-10754-7

Supplementary table 1. Overview of the geriatric assessment and outcome measures questionnaires and cut-off scores

Domains	Variables	Questionnaires	Score ranges	Cut-off points
**Geriatric assessment**				
Physical	Comorbidity	ACE 27	0-3	0 = None<br>1 = Mild<br>2 = Moderate<br>3 = Severe
	Intoxication	History of smoking	N.A.	Never<br>Former<br>Current
		History of alcohol consumption	N.A.	Never<br>Former<br>Current
	Nutrition	MUST	0-6	0 = Low risk<br>1 = Medium risk<br>≥2 = High risk
Functional	Mobility	TUG	N.A.	<13.5 = No restrictions<br>≥13.5 = Declined mobility
	Activities of Daily Living	Katz-ADL	0-6	<1 = No restrictions<br>≥1 = Restrictions
		Lawton-IADL	0-7	<1 = No restrictions<br>≥1 = Restrictions
	Fall risk	Fall risk	0-1	0 = No risk<br>1 = Risk
Psychological	Cognition	MMSE	0-30	>24 = Normal cognitive function<br>≤24 = Declined cognitive function
	Depression	GDS-15	0-15	<6 = No depression<br>≥6 = Depression
	Delirium risk	Delirium risk	0-5	0 = No risk<br>≥1 = Risk
Frailty	Frailty screening	GFI	0-15	<4 = Non-frail<br>≥4 = Frail
		G8	0-17	>14 = Non-frail<br>≤14 = Frail
**Outcome measures**				
Quality of Life	Swallowing Quality of Life	HNSW-QoL	Four questions 1-4	N/A (continuous variable)
			Total score 4-16	
			Total raw score 0-100	

Treatment related adverse events	Toxicity	CTCAE – D	0-5	N/A (continuous variable)

Legend: ACE = Adult Comorbidity Evaluation 27; MUST = Malnutrition Universal Screening Tool; TUG = Timed Up & Go; Katz-ADL = Katz Index of Activities of Daily Living; Lawton-IADL = Lawton Index of Instrumental Activities of Daily Living; MMSE = Mini Mental State Examination ; GDS-15 = Geriatric Depression Scale ; GFI = Groningen Frailty Indicator; G8 = Geriatric Screening Tool; HNSW-QoL = EORTC QLQ – H&N35 Swallowing domain; CTCAE-D = Common Terminology Criteria for Adverse Events – Dysphagia.

Hurtado-Oliva J., van der Laan H.P., de Vries J., Steenbakkers R.J.H.M., Halmos G.B., Wegner I (2024). Impact of Frailty on Post-Treatment Dysphagia in Patients with Head and Neck Cancer, Journal of Dysphagia, doi: https://doi.org/10.1007/s00455-024-10754-7

Supplementary figure 1. Flowchart diagram of patients cohort for analysis.

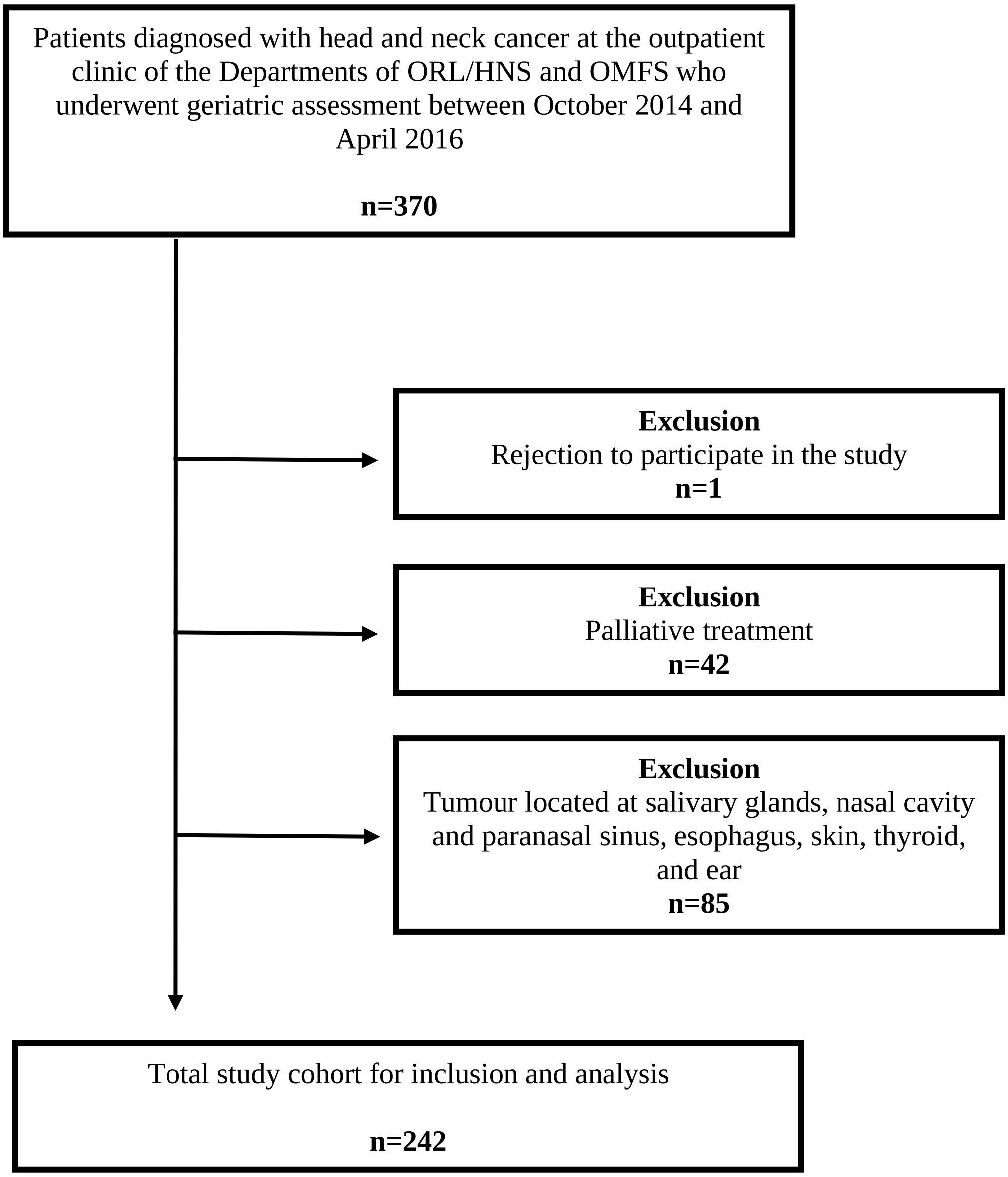

Legend: ORL/HNS = otorhinolaryngology/head and neck surgery; OMFS = oral and maxillofacial surgery.
